# Trustful conversations: a qualitative interview study on older patients’ experiences of the intervention *Proactive healthcare* in a Swedish primary care setting

**DOI:** 10.1017/S1463423623000427

**Published:** 2023-08-24

**Authors:** Åsa Sax, Magnus Nord, Elisabet Cedersund, Anna Olaison, Annette Sverker, Lisa Kastbom

**Affiliations:** 1 Primary Health Care Centre in Ljungsbro, and Department of Health, Medicine and Caring Sciences, Linköping University, Linköping, Sweden; 2 Department of Health, Medicine and Caring Sciences, Linköping University, Linköping, Sweden; 3 Primary Health Care Centre in Valla, and Department of Health, Medicine and Caring Sciences, Linköping University, Linköping, Sweden; 4 Department of Culture and Society, Linköping University, Linköping, Sweden; 5 Pain and Rehabilitation Center, and Department of Activity and Health, and Department of Health, Medicine and Caring Sciences, Linköping University, Linköping, Sweden; 6 Primary Health Care Centre in Ekholmen, and Department of Health, Medicine and Caring Sciences, Linköping University, Linköping, Sweden

**Keywords:** continuity, elder care teams, older patients, primary care, qualitative research, quality of care

## Abstract

**Aim:**

To explore older patients’ experiences of the intervention *Proactive healthcare for frail elderly persons.*

**Background:**

Previous research has indicated that continuity and good access to primary care can improve satisfaction in older people seeking care. However, little is known about the older patients’ experiences in taking part of interventions aiming to enhance the care.

**Methods:**

Individual interviews were conducted with 24 older patients who participated in the intervention *Proactive healthcare for frail elderly persons,* selected from nine Swedish primary care centres. Interviews were analysed using qualitative content analysis.

**Findings:**

Older patients’ experiences of the intervention involved five manifest categories: *Ways of naming the elder care team*, covering the older patients’ lack of understanding regarding their connection to the team, and the need for clarity on this and on how the specialised care provided differed from conventional care; *Availability*, indicating how older patients associated easy access and a direct telephone number with a team nurse available at certain times with a sense of security; *The importance of relations*, covering how patients appreciated continuity in their personal and professional conversations with staff; *A feeling of safety and trust*, stressing the value of older persons attach to being given enough time, to be listened to and being recognised as people; and *Finiteness of life*, which refers to the difficulty of having end-of-life conversations and the need for experienced staff with personal knowledge of the patients. The latent theme *Trustful conversations* was created to give a deeper meaning to the content of the categories.

Trustful conversations, created through good personal knowledge of patients and continuity of contact, engender a feeling of safety in older patients. Using elder care teams could result in a better quality of care, with increased satisfaction and feelings of security among patients, and a reduction in healthcare needs.

## Introduction

The world population is ageing and the proportion of older people in society is increasing, which puts a demand on most sectors of society, one of these being healthcare (Department of Economic and Social Affairs, 2002). The World Health Organization has defined healthy ageing as a process of maintaining functional ability to enable well-being in older age (Rudnicka *et al.*, 2020). To meet the needs of a growing and ageing population, and in order to make economic resources last, Sweden is in the process of developing care by focusing on and referring a larger proportion of healthcare to primary care (Nergårdh *et al.*, 2018). Similar changes are seen in other countries in Europe (WHO, 2015). In comparison with other European countries, Sweden has a good quality of healthcare overall. However, deficiencies regarding continuity of care, access to professionals and patient participation have been shown (WHO, 2015; Nergårdh *et al.*, 2018). According to Swedish law (Socialdepartementet, 2017), healthcare should be of good quality, efficient, accessible, person-centred and individualised (Nergårdh *et al.*, 2018).

Person-centred care (PCC) is one of the cornerstones of primary care in Sweden, as in many European countries and beyond (Socialstyrelsen, 2018; Ebrahimi *et al.*, 2021). The concept applies to all types of patients, and it is seen to be especially useful when there are more complex care needs, as is often the case in older people. The individuals’ needs, abilities and goals should be taken into account (Ekman *et al.*, 2011; Kogan *et al.*, 2016; Ebrahimi *et al.*, 2021), and it is essential that healthcare staff have personal knowledge of these factors (Ebrahimi *et al.*, 2021). Mutual communication characterised by trust and respect is an important quality of PCC (Coleman *et al.,* 2013; Ebrahimi *et al.*, 2021). Ebrahimi *et al.* (2021) conducted a systematic review of PCC in older people in out-patient settings. Four crucial key findings were presented, namely: knowing and confirming the patient as a whole person; conducting a tailored personal health plan; interprofessional teamwork and collaboration with and for older people and relatives; and building a person-centred foundation. This foundation is formed by reinforced access to the healthcare system and flexible organisation based on trusting relationships and authentic engagement (Ebrahimi *et al.*, 2021). The authors of the review believe that old age and frailty are factors that, for some, can affect the experience of accessibility in a negative way due to physical and/or cognitive impairment (Ebrahimi *et al.*, 2021). Tiilikainen *et al.* (Tiilikainen *et al.*, 2019) found that older people described difficulties in gaining access to healthcare services and that these factors were closely linked to their sense of autonomy in everyday life. Furthermore, the study showed that they experienced a lack of recognition of their own personhood and individual needs from those within the services (Tiilikainen *et al.*, 2019). In a systematic review from 2020 (Frost *et al.*, 2020), effective components of primary and community management of complex conditions in older people were identified. It was suggested that integrated models of care, such as patient education, self-management, interprofessional collaboration and professional support, contribute to positive effects (Frost *et al.*, 2020). According to d’Amour *et al.*, there is disagreement about the degree to which the patient is considered to be a part of the team/collaboration (D’Amour *et al.*, 2005).

### The intervention Proactive healthcare for frail elderly persons

This study is a follow-up to the intervention *Proactive healthcare for frail elderly persons*, a study which involved five municipalities and 19 primary care centres (nine intervention practices and 10 control practices) in the southeast of Sweden from 2017 to 2019 (Marcusson *et al.*, 2019; Nord *et al.*, 2020, 2021). The intervention included 1,600 patients aged over 75 years. These patients were selected with a prediction model which estimates the risk of hospitalisation from electronic medical records (Marcusson *et al.*, 2020). Age and healthcare use were the most important variables in the model. Half of the patients selected were offered to participate in the intervention at their primary care centres and the other half received usual care at matched control centres. The intervention involved a specialised *elder care team* (in Swedish: *äldremottagning*) at each primary care centre, consisting of a nurse and a physician who made a comprehensive geriatric assessment (CGA) of each patient by using the Primary Care Assessment Tool for the Elderly (Nord *et al.*, 2020, 2021). The assessment tool was created for the intervention and included physical, social and psychological items. Patients were asked about their preferences for care and about thoughts concerning their future. There was no standard treatment after the assessment, and the teams had different ways of presenting their work model to the patients. The elder care teams were encouraged to provide continuity, facilitate accessibility and individualise treatment and follow-up (Nord *et al.*, 2020). In this intervention, the frailty model developed by Rockwood and Mitinski, and applied in the Clinical Frailty Scale (Rockwood *et al.*, 2005), was used. After the end of the intervention study in 2019, the elder care teams at the intervention centres continued their work model and similar teams were subsequently implemented in the region. This means that the participants in our study, even after the introduction of the intervention, had regular contact with their elder care teams. The team was thus the main care contact at the primary care centre, so the phenomenon of elder care teams, therefore, was well known to the participants.

A previous qualitative interview study had taken place in an early phase of the intervention and focused on experiences of involvement in care from the perspective of the older patients. The findings from this study suggested that participation in care, as described by the older persons, is interwoven with a desire for an understanding of the structure of the care system on an overarching level as well as on an interpersonal and personal level (Olaison *et al.*, 2021). However, the participants did not explicitly refer very much to the intervention, which could be because the interviews were conducted early after its onset, when they had not had enough time to become familiar with it. This gave rise to the idea of the present follow-up study. When implementing new procedures in healthcare, it is crucial to explore, in addition to the effectiveness of the intervention, the experiences and feelings of those taking part of the implementation as a variable of revaluation in this particular intervention (Nord *et al.*, 2021).

The aim of this study was to explore older patients’ experiences of the intervention *Proactive healthcare for frail elderly persons.*

## Material and methods

### Participants and interviews

Inclusion criteria were that the participant should be a patient at any of the nine primary care centres that had participated in the intervention *Proactive healthcare for frail elderly persons* (Marcusson *et al.*, 2019; Nord *et al.*, 2020, 2021), be Swedish-speaking and accept that the interview would be recorded. Participants were recruited through a data search of the Care Data Warehouse (in Swedish: *Vårddatalagret*), which contains all healthcare contacts in Region Östergötland (the county council). The data search selected, from the primary centres that had participated in the intervention, 100 patients who had the most frequent contacts with the healthcare system. From this selection, every third patient was asked to participate. The responsible nurses at the primary care centres had good knowledge of their patients and were asked to confirm if the selected patients still had regular contact with the elder care teams. The nurses also assessed if the selected patients were able to participate in a telephone interview about their experiences, which included an estimation of the patients’ cognitive capacity. Thus, only those patients who were assessed by the nurses to be cognitively capable to take part were asked to participate in the present study. Since contact over the telephone is common in the elder care teams, the nurses’ assessments were based on this knowledge of the patients. All three areas of the region, and both urban and rurally located primary care centres, were represented, to ensure a wide variety of participating patients (Patton, 2015). Of the 37 individuals who were asked to participate in the study, 24 agreed to take part. Frequently mentioned reasons for refraining from participating were poor health and having participated in several studies previously.

An interview guide was constructed, consisting of open questions concerning each patient’s everyday life, views on participating in the intervention *Proactive healthcare for frail elderly persons*, experiences of being taken care of at the primary care centre, and how they related to the future and end-of-life (EOL) issues as the intervention had an ambition to facilitate discussions about these matters. Clarifying questions were asked (Patton, 2015). These follow-on questions were asked to minimise the risk of obscurity in any of the statements made by the participant.

The interviews were performed approximately one year after the intervention study had ended. The nine intervention primary care centres that had implemented the model still had their teams up and running when the interviews were held. Four of the researchers, all with experience in qualitative research but from different scientific disciplines, such as health, ageing and social work research, conducted the interviews. One of the interviewers was a general practitioner, and two were social workers. In total, 24 participants were interviewed individually. An overview of the participating patients is presented in Table 1. Common characteristics were old age, multimorbidity and frailty to varying extent. Due to the COVID-19 pandemic, the interviews were carried out by telephone. None of the informants dropped out at any phase of the study. The interviews were conducted in 2020 and were digitally recorded and transcribed verbatim.

**Table 1.** Demographics and characteristics of study sample (*n* = 24)

Age mean (range)	84 years (75 – 92)
Gender men/women (*n*)	33% (8)/67% (16)
Cohabitant/living alone (*n*)	37.5% (9)/62.5% (15)
Having children yes/no (*n*)	79% (19) /21% (5)
Swedish-born yes/no (*n*)	100% (24) /0% (0)

### Analysis

The interviews were analysed through qualitative content analysis with no predetermined categories or themes (Graneheim and Lundman, 2004; Patton, 2015). The analysis was performed using the following seven steps: (1) the transcribed interview was read through to obtain an overall impression and to gain a broad understanding; (2) segments of the texts dealing with the aim of the study were identified, and meaning units were constructed; (3) the meaning units were condensed and abstracted to codes; (4) the codes were compared and sorted into categories; (5) the categories were compared with the entire interview to make sure that the interpretation was consistent and coherent with the text as a whole; (6) the categories were compared to avoid overlapping, and content descriptions were developed; and (7) quotations were used to exemplify the categories. One latent theme was created to give a deeper meaning of the manifest categories in the analysis (Graneheim and Lundman, 2004). Examples of the steps of the analysis are shown in Table 2.

**Table 2.** Examples of the steps of the analysis using qualitative content analysis

Meaning unit	Code	Category
‘… It is easier to get in touch because there is always a nurse in the elder care team in the morning for an hour, so you know that she will answer. You reach her directly and that is good. It feels safe’.	Availability	Availability
‘You never get the same physician every time like it used to be … Yes I never get to know the physician, and the physician never really gets to know me … like it used to be before, it was much better a few years ago’.	Discontinuity	The importance of relations
	Patient-physician relationship	
‘I take one day at a time. I don’t think about how it will be in a year, in five years or so. No. I don’t. You know I don’t think along those lines, but have the approach that when that day comes, I won’t be able to protest, but will just have to come along’.	Live for the day	Finiteness of life
	End-of-life	

The preliminary categories were mainly coded by the first and last authors. The tentative categories were then discussed and revised by all the researchers. As part of the reflexivity process, the categories were validated by supplementing and contesting each other’s readings and preunderstandings (Malterud, 2001). To ensure the anonymity of the participants in the study, the background characteristics of the interviewees were left out when presenting quotes.

## Results

**Figure 1.**
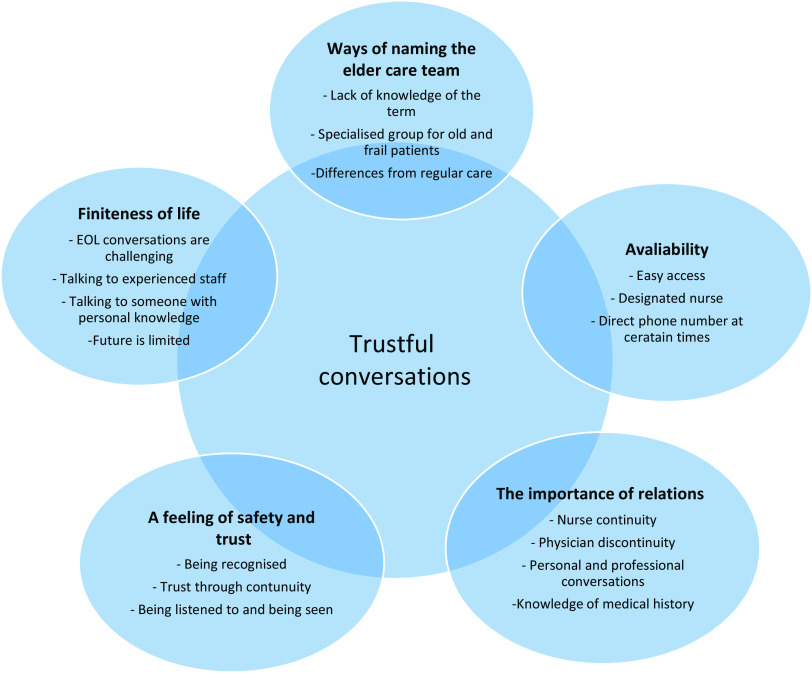
Five manifest categories with examples describing the participants’ experiences of the elder care team at the primary care centre. The latent theme *Trustful conversations* was created to give a deeper meaning to the content of the manifest categories.

When analysing the data through qualitative content analysis (Graneheim and Lundman, 2004), five manifest categories describing the patients’ experiences of the intervention *Proactive healthcare for frail elderly persons* were seen, namely: *Ways of naming the elder care team; Availability; The importance of relations; A feeling of safety and trust;* and *Finiteness of life*. One latent theme was created to give a deeper meaning to the content of the manifest categories, that is, *Trustful conversations* (see Fig. 1).

### Ways of naming the elder care team

The interviewees had various ways of naming the elder care team at the primary care centres. Some had not even heard of such a team, even though they were connected to it through the intervention *Proactive healthcare for frail elderly persons.* Those who were unfamiliar with the term ‘elder care team’ talked about it as being the ordinary primary care centre, although they seemed to have been in contact with one or several designated nurses with a connection to the team. Some mentioned a specific telephone number that was used to facilitate the contact with their primary care centre. This confirmed that they had actually been part of the intervention despite their lack of knowledge of the term ‘elder care team’.

‘*I have no idea what that* [elder care team] is. I have never heard of an elder care team. When I have ailments or have an injury, then I call the primary care centre XX [name of the primary care centre] and then I get an appointment and go there to get it fixed’.

(Interview no. 9:82-year-old man.)

Some participants had been in contact with their designated nurse, even though they were not aware that the nurse was part of a team. To some, the meaning of an elder care team seemed very clear, with no uncertainties, and these participants were obviously aware of the term. For them, there was a clear understanding that the care provided by the team differed from the regular care provided by the primary care centre and that they were a part of a selected group of individuals.

‘*I have been called to a special group down here* [the primary care centre] *and had an interview and all about my situation. And have also been called a few times to this group for patients with multimorbidity*’.

(Interview no.11:79-year-old man.)

### Availability

The method of reaching the designated nurse differed somewhat between the primary care centres. Some had a special number for the elder care team, while others mentioned using the ordinary telephone number. Easy access and a direct way of making contact with the nurse in the team were appreciated by the interviewees. They knew what number to call for a designated nurse and during what times this nurse was available. Many seemed to have called the direct number, while others had contacted the primary care centre through the regular telephone queueing system. By talking to the designated nurse, the participants were able to book a physician consultation, ask for a prescription of drugs, or resolve situations with the nurse. This experience of easy access gave a sense of security.

‘*… It is easier to get in touch because there is always a nurse in the elder care team in the morning for an hour, so you know that she will answer. You reach her directly and that is good. It feels safe*’.

(Interview no. 5:86-year-old woman.)

Using the ordinary telephone queueing system required the older patients to press buttons to make several choices, and this was found difficult by them; thus, there was a sense of relief when they did not have to go through it and could instead use the direct number for the nurse in the elder care team.

‘*I am so inexperienced with all this, new phones and data and all this, so I think it is troublesome to have to keep on pressing … yes, press this and that, press square and all this. It is a lot to do before getting in touch and talking to someone at the primary care centre. I think most older people find this troublesome*’.

(Interview no. 15:92-year-old woman.)

### The importance of relations

Participants described the value of continuity through long-lasting patient–physician relations. Such relations could be interpreted as a combination of a professional and a more personal relationship. According to several of the interviewees, such patient–physician relations no longer exist. The lack of such long-term relations and lack of continuity regarding the physician at the primary care centre, leading to discontent and the repetition of one’s medical history at physician consultations, were described as tiresome.

‘*You never get the same physician every time like it used to be… Yes, I never get to know the physician, and the physician never really gets to know me … like it used to be before, it was much better a few years ago*’.

(Interview no. 5:86-year-old woman.)

According to the participants, a good and continuous relationship with the nurse in the elder care team could lead to more trusting and personal conversations regarding health issues, but also with regard to everyday matters.

‘*The thing I appreciate is that one [the nurse] takes a little time, one engages in questions and situations. Not only checks them off but … I have a good relationship with XX [name of the nurse connected to the elder care team] and I think it has … yeah, I am very positive about this approach and it has worked so far anyway*’.

(Interview no. 17:82-year-old man.)

### A feeling of safety and trust

In order for patients to feel safe and cared for, continuity in healthcare was described as important. The continuity shaped by the possibility of contacting a designated nurse in the elder care team provided this sense of safety and trust despite the discontinuity experienced in relation to physicians. To be recognised over the telephone or in person was a positive aspect of being involved with the team. For some, there was a belief that this continuity led to less need for physician consultations, since issues could be resolved through contact with the nurse, who consulted the physician when needed.

‘*Yes, it is easier to get in touch, I guess nowadays and I have got a nurse there who I have been in contact with quite recently on occasions and then you … then it is easier to talk to them also. When I come to the ordinary clinic you never know how many physicians or nurses you will come into contact with*’.

(Interview no. 20:85-year-old woman.)

Other aspects that were described as improvements brought about by being connected to the team included the feeling of being heard and believed in, being given enough time to talk, and having matters explained properly. In previous contacts with healthcare services, patients had experienced being rushed and feelings that they were less important because of being older.

‘...*that there is someone there who listens and believes what one says. That they listen to what you say and do not only assume that because you have some kind of diagnosis, that does not have to be the reason it hurts somewhere else*’.

(Interview no.7:81-year-old woman.)

### Finiteness of life

When the participants were asked about EOL issues, some mentioned that they had talked about such matters with their children. Others wanted to talk to a professional, such as a physician, a nurse, a therapist or a priest, while others did not wish to discuss these matters at all. For those who did want to talk about EOL, having faith in the person they were talking to was important. Having these kinds of conversations with older or, at least, more experienced staff was preferred. Such matters did not seem to have been discussed in the context of the elder care team.

[On who to talk to about EOL questions] ‘*Good thing. I don’t know. It is probably up to me, but then I would like to pick it up with someone who I think is older. An experienced nurse, some kind of therapist or suchlike*’.

(Interview no. 9:82- year-old man.)

For those who did not want to have these kinds of conversations, the subject itself and the patient’s limited future could be difficult to think about, and also, there could be a reluctance to make plans for the last days of life. Protecting family members and not wanting to bother others with one’s thoughts were reasons to avoid talking about EOL issues.

[On conversations about EOL and with whom to speak about this] ‘*Well, it is not that easy. I think for myself, if I was to talk to someone, yes, it has to be someone … oh no, I would not expect this of any person. It has to be the ones who know me well*’.

(Interview no. 12:92-year-old woman.)

When talking about views regarding the future, it was described as a short period of time ahead. There might be short-term plans, such as holiday trips or seeing grandchildren. For others, the future was something they no longer wanted to talk about and there was a wish for life to end.

Living for the day and being grateful for every day were recurrent themes in the interviews. Being able to take care of oneself and continue to live at home, rather than moving to a nursing home, were important matters.

‘*I take one day at a time. I don’t think about how it will be in a year, in five years or so. No. I don’t. You know, I don’t think along those lines, but have the approach that when that day comes, I won’t be able to protest, but will just have to come along*’.

(Interview no. 8:85-year-old woman.)

The older patients had faith in the healthcare system and presumed that they would be given help when they deteriorated and their ability to take care of themselves was lost.

‘*I feel trust in the healthcare, that they will help to make the transition as good as possible. That is what I hope for*’.

(Interview no. 11:79-year-old man.)

### Trustful conversations

When conducting the analysis, a latent theme was created to give a deeper meaning to the content of the categories, namely the importance of having trustful conversations between the older person and the healthcare staff. Even though there was sometimes a lack of knowledge of the name ‘elder care team’, there seemed to have been contacts with the team’s nurse and trustful conversations about personal matters, which were described as valuable for the participants.

Easy access to the team provided a foundation for building safety and trust in the care offered, and this opened the way for conversations. Having contact with healthcare staff who had personal knowledge of the patients contributed to more direct conversations about any current issues and also provided an opportunity for more personal matters to be expressed. Some patients stated that they missed former physicians who had had good personal knowledge of them and with whom all matters, such as questions around preferences regarding the EOL, could be discussed. This no longer occurred, according to these patients.

To be recognised in person or over the telephone made the participants feel important and seen. Being given enough time to speak, being listened to, and not feeling rushed or marginalised for being older were also important in engendering a sense of mutual respect and building a foundation for trustful conversations between the older person and the healthcare staff. The preconditions for trustful conversations were dependent on the staff’s personal knowledge of the older person. There did not seem to have been communications about EOL in the context of the intervention, and the willingness of the older people to discuss questions concerning the last days of life differed to a great extent. Trustful conversations with their children or other family members about both practical and emotional matters regarding EOL occurred. Participants explicitly expressed that they wished to have these kinds of conversations with professionals and for the professionals to show interest in personal matters as well as medical issues.

## Discussion

### Statement of principal findings

This study revealed five manifest categories describing the older patients’ experiences of the elder care teams at the primary care centres, namely: *Ways of naming the elder care team*, indicating the older patients’ lack of understanding of their connection to the team, but also the need for clarity regarding the team’s specialism in caring for older persons and the differences between this care and the regular care offered by the primary care centre; *Availability*: referring to the sense of security that older patients gained from easy access to, and a direct telephone number for a designated nurse who was available at certain times; *The importance of relations*: stressing the value of the patients attached to continuity in personal and professional conversations with staff, where sometimes nurse continuity could partly compensate for physician discontinuity; *A feeling of safety and trust*: covering the older persons’ need to be given enough time to speak, to be listened to and to be recognised as a person, all valuable components of the care; and *Finiteness of life*: pointing to the difficulty patients could experience in having EOL conversations, and the need for experienced staff with personal knowledge of the patients. The latent theme *Trustful conversations* was created to give a deeper meaning of all the five manifest categories describing the patients’ experiences of the elder care team.

### Strengths and weaknesses of the study

One strength of this study was the involvement of researchers from various scientific disciplines during all stages, from performing the interviews to producing the finished article. The interviews were at all times performed by someone without personal knowledge of the participant, working at a different primary care centre if clinically active. By involving those with different scientific backgrounds and professions, the method and approach to interviewing probably varied to some extent, resulting in a wider variety of subject matter being highlighted during the sessions and thus giving a greater depth to the material. The interviews were conducted approximately one year after the intervention study had ended, but the participants still had an active relation with their elder care teams during the time when the interviews were conducted. This gave the participants enough time to reflect and take part of the elder care team to have a good apprehension about the form of care in comparison to the regular care provided by the primary care centre. On the other hand, certain aspects of the intervention, for example, the multimodal assessment (CGA), were carried out in 2017, and participants may have had difficulty in relating to this aspect because of the delay.

During the intervention, the primary care centres were free to use different approaches regarding the team, such as how patients should contact the team nurse; there was also a slight variation in the participating professionals, apart from the physician and the nurse, and differences in the frequency of using the term ‘elder care team’. This may have affected patients’ experiences of their interactions with the team and thus led to variations in the responses.

The respondents were recruited from both urban and rurally located primary care centres in all three areas of the county studied, which gave a broad variety of participants, supporting the generalisability of the findings. All the respondents were born in Sweden, with Swedish as their only main language. This probably led to fewer linguistic misunderstandings but should be seen as a weakness overall, as it meant that the immigrant population in the society were not represented.

Due to the COVID-19 pandemic, all the interviews were performed over the telephone. This may have resulted in more honest answers, as there was no need for participants to be face-to-face with the person asking the questions, thus reducing the discomfort when talking about sensitive issues through social distance (Sturges and Hanrahan, 2004; Oltmann, 2016). However, it may also have led to less dynamic conversations, as the non-verbal aspect of the conversation was therefore missing. The research group thoroughly discussed possible advantages and disadvantages of having EOL conversations over the telephone in an early phase of the planning of the study. When evaluating the initial interviews performed by the three interviewers, we were all agreed that also sensitive topics, such as questions concerning the future and EOL issues, could be discussed when interviewing on the telephone, provided that the interviewer is perceptive and sensitive to any possible reaction of the participant. The participants had been informed, before accepting to participate in the study, that the interviews included questions about EOL. Since the participants still had connections to the elder care teams and were well known to the responsible nurses at their teams, any questions or concerns that could arise after the interviews were possible to discuss afterwards. The researchers who performed the interviews were experienced interviewers, with previous experiences in interviews focusing on EOL issues. As a result, during each interview, there was an increased perception of the participant’s clarity and sensitivity to not wanting to talk further about the subject.

### Findings in relation to other studies

We found that easy and direct access to professionals was of great importance and contributed to a feeling of being safe and having trust in the healthcare system. This correlates with the results from a review by Lawless *et al.* (Lawless *et al.*, 2020), who identified that the ability to access healthcare was critical for older people. Problems related to physical access led to anxiety, especially when patients were required to wait a long time for appointments.

In the present study, being able to make contact in a quick and easy manner and speak to a familiar person was perceived as positive, reassuring and time-efficient. During the intervention, the primary care centres were given the option of providing a direct telephone number for patients to make contact with a nurse connected to the elder care team who was available at specific times or referring the participants to the regular telephone queueing system to contact their designated nurse. This probably affected the experience of accessibility in a negative way for those who still had to go through the queueing system and choose options by pressing buttons.

In the intervention *Proactive healthcare for frail elderly persons*, a significant reduction in hospital care days in the intervention group was seen. Furthermore, the visits to primary care did not increase in numbers, and there was no difference in mortality in comparison with the control group (Nord *et al.*, 2021). This is supported in a review by Huntley *et al.* (Huntley *et al.*, 2014), who have suggested that continuity and access to primary care can reduce unscheduled care use. In general, older age and multimorbidity were both associated with more visits to the emergency department and acute admissions to hospital (Huntley *et al.*, 2014). In another review, continuity and adequate physician supply were found to be of great importance to achieving low rates of avoidable hospitalisation (van Loenen *et al.*, 2014). Furthermore, continuity was defined as a long-term relationship with a primary care provider (van Loenen *et al.*, 2014). A study on primary care in a Swedish context showed that active listing of patients to physicians and more consultations at a primary care centre, thus giving continuity, led to a reduced number of days of hospitalisation (Ranstad *et al.*, 2018).

In the study, we found that easy access to the nurse answering the call, in combination with the nurse’s personal knowledge of the patient, can lead to more open conversations about health issues as well as life in general. Continuity is essential to gaining this personal knowledge. This is supported by Nutting *et al.* (Nutting *et al.*, 2003) who have shown that continuity appears to increase the satisfaction with the care given, particularly for more vulnerable people, such as older people. The present study showed that participants described their experiences with the elder care team as positive owing to the continuity provided by having a designated nurse. Having personal knowledge of the patient is likely to allow the nurse to give good advice over the telephone and to determine whether the patient needs a physician’s appointment and if so, in what time frame. This may contribute to optimising the number of appointments with the physician by prioritising the patients in most need. Having a good knowledge of the patient’s habitual status facilitates assessment and helps to determine the urgency of the need for treatment. Metzelthin *et al.* (Metzelthin *et al.*, 2013) found that the use of an interdisciplinary team led to a better understanding of the concerns, wishes and problems of frail older people. Clearly defined roles and tasks within the team led to more efficient healthcare delivery, and a good relationship with the healthcare professionals was perceived as a safety net by the older people (Metzelthin *et al.*, 2013).

We found that with continuity and personal knowledge, trust can be achieved. Being given enough time to talk and time to explain issues seemed to be of importance in achieving trust and a feeling of safety. When patients have to repeat their medical history to new staff, valuable time is being wasted, and the patients appeared to experience a sense of being rushed and not being as important as younger patients. Similar results were found in a previous study, where older people expressed a wish to be taken seriously, appreciated and given time and attention by their physician (Metzelthin *et al.*, 2013). Perhaps more time should be set aside for physician appointments with older people in general in order to provide a better quality of care. This should benefit not only the patient, but also the entire healthcare system.

Despite good continuity by the nurses, there seemed, according to the participants, to have been a lack of continuity among the physicians. In some ways, this could be compensated for by the nurse having a good knowledge of the patient, as discussed above. However, the patients clearly did not appreciate this discontinuity, and this may contribute to a more negative experience overall, something which has also been shown previously in a Finnish primary care context (Raivio *et al.*, 2014).

To enable conversations about subjects that, for some, are difficult to approach, a confiding relationship is essential. Sharp *et al.* found that the majority of frail elderly people wanted to discuss EOL care, but were not given the opportunity (Sharp *et al.*, 2013). In the present study, EOL care was not often discussed. However, in cases where such issues were talked about, the older patients preferred to discuss them with a person they had trust in, for example, experienced healthcare staff or a family member. The opportunity for such discussions relies on physicians getting to know their patients better and it cannot be replaced by continuity of contact with the nurse, since medical planning and decisions about life-resuscitating care can only be made by a physician. The feeling of discontinuity or lack of physician presence was also found in a Swedish nursing home context when exploring opinions on EOL care planning among family members of older persons (Kastbom *et al.*, 2020). The nurse was felt to be more present and available, while the physician was described as absent (Kastbom *et al.*, 2020). Kastbom *et al.* have further highlighted the importance of having a team approach when planning the care of older persons, with the nurse both initiating the care planning and following up to confirm the content of the documented plan with the patient and family members (Kastbom *et al.*, 2019).

If healthcare can provide both physician and nurse continuity, along with easy access to these professionals, to bring comfort and trust through elder care teams, proactive planning for the care of the patient is facilitated. Personal knowledge of the patient is of importance, in order to provide a good quality of care and to make plans for future events, to ensure that the care is optimised and accords with the patient’s own wishes.

### Implications of the study

The findings of this study have implications for staff caring for older, frail patients, especially in a primary care context. The findings stress the importance of trustful conversations that are created through good personal knowledge of the patients and continuity of contact with professionals, which results in a feeling of safety and contentment with primary care for the older patients. The results can be used to stress the importance of continuing and further developing the use of the elder care teams using CGA and establishing individualised care plans. Improving continuity among physicians and ensuring enough time is given at each appointment with older, frail patients should result in a better quality of care, increased satisfaction and a feeling of security among patients, and possibly a further reduction in healthcare needs.
